# Inferring the role of transcription factors in regulatory networks

**DOI:** 10.1186/1471-2105-9-228

**Published:** 2008-05-06

**Authors:** Philippe Veber, Carito Guziolowski, Michel Le Borgne, Ovidiu Radulescu, Anne Siegel

**Affiliations:** 1 Centre INRIA Rennes Bretagne Atlantique, IRISA, Rennes, France; 2 Université de Rennes 1, IRISA, Rennes, France; 3 Université de Rennes 1, IRMAR, Rennes, France; 4 CNRS, UMR 6074, IRISA, Rennes, France

## Abstract

**Background:**

Expression profiles obtained from multiple perturbation experiments are increasingly used to reconstruct transcriptional regulatory networks, from well studied, simple organisms up to higher eukaryotes. Admittedly, a key ingredient in developing a reconstruction method is its ability to integrate heterogeneous sources of information, as well as to comply with practical observability issues: measurements can be scarce or noisy. In this work, we show how to combine a network of genetic regulations with a set of expression profiles, in order to infer the functional effect of the regulations, as inducer or repressor. Our approach is based on a consistency rule between a network and the signs of variation given by expression arrays.

**Results:**

We evaluate our approach in several settings of increasing complexity. First, we generate artificial expression data on a transcriptional network of *E. coli *extracted from the literature (1529 nodes and 3802 edges), and we estimate that 30% of the regulations can be annotated with about 30 profiles. We additionally prove that at most 40.8% of the network can be inferred using our approach. Second, we use this network in order to validate the predictions obtained with a compendium of real expression profiles. We describe a filtering algorithm that generates particularly reliable predictions. Finally, we apply our inference approach to *S. cerevisiae *transcriptional network (2419 nodes and 4344 interactions), by combining ChIP-chip data and 15 expression profiles. We are able to detect and isolate inconsistencies between the expression profiles and a significant portion of the model (15% of all the interactions). In addition, we report predictions for 14.5% of all interactions.

**Conclusion:**

Our approach does not require accurate expression levels nor times series. Nevertheless, we show on both data, real and artificial, that a relatively small number of perturbation experiments are enough to determine a significant portion of regulatory effects. This is a key practical asset compared to statistical methods for network reconstruction. We demonstrate that our approach is able to provide accurate predictions, even when the network is incomplete and the data is noisy.

## Background

A central problem in molecular genetics is to understand the transcriptional regulation of gene expression. A transcription factor (TF) is a protein that binds to a typical domain on the DNA and influences transcription. The effect of this TF can be either a repression or an activation of transcription depending on the type of binding site, the distance to coding regions, or on the presence of other molecules. Finding which gene is controlled by which TF is a reverse engineering problem, usually named *network reconstruction*. This question has been approached over the past years by various groups.

A first approach to achieve this task is to collect the information spread in the primary literature. Following this idea, a large number of databases that take protein and regulatory interactions from the literature and curate them have been developed [1-5]. For the bacteria *E. coli*, RegulonDB is a dedicated database that contains experimentally verified regulatory interactions [6]. For the budding yeast (*S. cerevisiae*), the Yeast Proteome Database contains a large amount of regulatory information [7]. In this latter case, however, the amount of available information is not sufficient to build a reasonably accurate model of transcriptional regulation. Databases with regulatory knowledge extracted from the literature are, nevertheless, an unavoidable starting point for network reconstruction.

The alternative to a literature-curated approach is a data-driven approach. This approach is supported by the availability of high-throughput experimental data including microarray expression analysis of deletion mutants (simple or more rarely double non-lethal knockouts), over expression of TF-encoding genes, protein-protein interactions, protein localisation, or ChIP-chip experiments coupled with promoter sequence analysis. We may cite several classes of methods that use these kinds of data, such as correlation, mutual information or causality studies, Bayesian networks, path analysis, information-theoretic approaches, and ordinary differential equations [8-10].

In short, most available approaches so far are based on a probabilistic framework which defines a probability distribution over the set of models. The reconstructed network is then defined as the most likely model given the data. Such an optimization problem is usually non convex, and finding a global optimum cannot be guaranteed in practice. Existing algorithms report a local optimum which should be interpreted with care: errors can appear and no consensual model may be produced.

As an illustration, special attention has been paid to the reconstruction of *S. cerevisiae network *from ChIP-chip data and protein-protein interaction networks [11]. A first regulatory network was obtained with promoter sequence analysis methods [12,13], yet, some undetected transcriptional regulatory motifs were proposed using non-parametric causality tests [14]. Moreover, Bayesian analysis also identified new regulatory modules for this network [15,16]. Thus, the results obtained with the different methods do not coincide and a fully data-driven search is in general subject to over-fitting and not fully reliable [17].

In regulatory networks an important and non-trivial physiological information is the regulatory role of TFs as inducer or repressor, also called *the sign of the interaction*. This information is needed if one wants to know, for instance, the physiological effect of a change caused by external conditions or the effect of a perturbation on the TF. While this can be achieved for one gene at a time with (long and expensive) dedicated experiments, probabilistic methods such as Bayesian models [18] or path analysis [19,20] are capable of proposing models from high-throughput experimental data. However, as for the network reconstruction task, these methods are based on optimization algorithms that compute an optimal solution with respect to an interaction model.

In this paper, we apply formal methods to compute the sign of interactions in networks that have an available topology. By doing so, we also validate the topology of the network. Roughly, we use expression profiles to constrain the possible regulatory roles of TFs, and we report those regulations that are assigned the same role in all feasible models. Thus, we over-approximate the set of *feasible *models, and then look for *invariants *in this set. A similar idea was applied in [21] to check the consistency of gene expression assays. However, we use a deeper formalisation and stronger algorithmic methods to achieve the inference task.

Different sources of large-scale data are exploited in this study: gene expression arrays, which provide information on the interaction signs; and ChIP-chip experiments, which provide the topology of the regulatory network when not available.

The main tasks we address are the following:

1. Building a formal model of regulation for a set of genes that integrates information from ChIP-chip data, sequence analysis, and literature annotations.

2. Checking its consistency with expression profiles on perturbation assays.

3. Inferring the regulatory role of TFs as inducer or repressor if the model is consistent with expression profiles.

4. Isolating ambiguous pieces of information if it is not.

The Results section is organised as follows. We first introduce the mathematical framework which is used to define and to test the consistency between expression profiles and transcriptional networks. Then, we apply our algorithms to address three main issues:

• Analysis of the dependence between the number of available observations and the number of inferred regulations. In the case where all genes are observed, we prove that at most 40.8% of *E. coli *network can be inferred and that 30 perturbation experiments are enough to infer 30% of the network on average. In the case of missing observations, we estimate how the proportion of unobserved genes affects the number of inferred regulations.

• Illustration and validation of our method on the transcriptional network of *E. coli*, obtained from RegulonDB [6], with a compendium of expression profiles [9,22].

• Execution of our inference algorithms over the *S. cerevisiae *transcriptional network. We inferred, for small scale subnetworks, more than 20% of the roles of regulations. For more complex networks, we detected and isolated inconsistencies (ambiguities) between expression profiles and a significant part of the model (15% of all the interactions).

## Results

### Detecting the role of a regulation and validating a model

Our goal is to determine the regulatory role of a TF on its target genes by using expression profiles. Let us illustrate our purpose with a simple example.

We suppose that we are given the topology of a network (this topology can be obtained from ChIP-chip data or any computational network inference method). In this network, let us consider a node *A *with a single predecessor. In other words, the model tells us that the protein *B *acts on the expression of the gene coding for *A *and no other protein acts on *A*.

**Figure 1:**
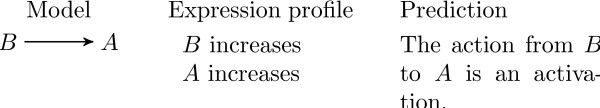
Illustration of the simple inference rule.

Independently, we suppose that we have several gene expression arrays at our disposal. One of these arrays indicates that *A *and *B *simultaneously increase during a steady state shift experiment. Then, *common sense *tells us that *B *must have been an activator of *A *during the experiment. More precisely, protein *B *cannot have inhibited gene *A *since they both have increased. Consequently, we say that the model *predicts *the sign of the interaction from *B *to *A *as positive (see Fig. 1).

**Figure 2:**
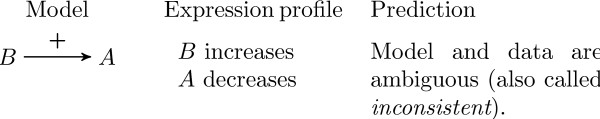
A simple case of inconsistency between some data and a model.

This naive rule is actually used in a large class of models; we will call it the *naive inference rule*. When several expression profiles are available, the predictions of the different profiles can be compared. If two expression profiles predict different signs for a given interaction, there is an *ambiguity *or *inconsistency *between data and model (see Fig. 2). Then, the ambiguity of the regulatory role can be attributed to three factors: (1) a complex mechanism of regulation, the role of the interaction depends on the state of the system; (2) a missing interaction in the model; (3) an error in the experimental source. This simple strategy is implemented in the Algorithm 1.

Let us consider now the case when *A *is activated by two proteins *B *and *C*. No more natural deduction can be done when *A *and *B *increase during an experiment since the influence of *C *must be taken into account. A *model *of interactions between *A*, *B*, and *C *has to be proposed. Probabilistic methods estimate the most probable signs of regulations that fit with the theoretical model [18,23].

Our point of view is different; we introduce a *basic rule *that shall be checked by each interaction in the model. This rule tells us that **any variation of *A *must be explained by the variation of at least one of its predecessors**. In previous papers, we introduced a formal framework to justify this basic rule under some reasonable assumptions. We also tested the consistency between expression profiles and a graphical model of cellular interactions. This formalism will be introduced here in an informal way; its full justification and extensions can be found in the references [24-27].

**Figure 3:**
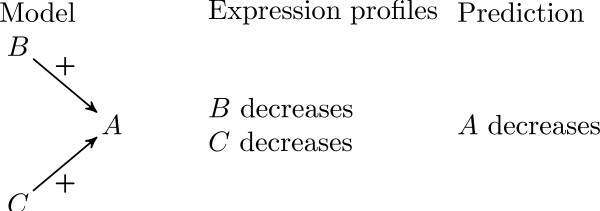
Illustration of a prediction.

In our example, the basic rule means that if *B *and *C *activate *A*, and both (*B *and *C*) are known to decrease during a steady state experiment, *A *cannot be observed as increasing. Then *A *is *predicted *to decrease (see Fig. 3). More generally, we apply the rule as a constraint for the model, we write constraints for all the nodes of the model, and we use several approaches in order to solve the system of constraints. From the study of the set of solutions, we deduce which signs are surely determined by these rules. Then, we obtain *necessary conditions *on the signs instead of the *most probable signs *given by probabilistic methods.

### A formal approach

Consider a system of *n *chemical species {1,...,*n*}. These species interact with each other and we model these interactions using an *interaction graph G *= (*V, E*). The set of nodes is denoted by *V *= {1,...,*n*}. There is an edge *j *→ i ∈ *E *if the level of species *j *influences the production rate of species *i*. Edges are labelled by a sign {+, -} which indicates whether *j *activates or represses the production of *i*.

In a typical stress perturbation experiment a system leaves an initial steady state following a change in control parameters. After waiting long enough, the system may reach a new steady state. In genetic perturbation experiments, a gene of the cell is either knocked-out or over-expressed; perturbed cells are then compared to the reference. Our approach relies on the signs of the variations in expression or activity of the species in the network. Let us denote by *sign*(*X*_*i*_) ∈ {+, -, **0**} the sign of the variation of species *i *during a given perturbation experiment, and by *sign*(*j *→ *i*) ∈ {+, -} the sign of the edge *j *→ *i *in the interaction graph.

Let us fix species *i *such that there is no positive self-regulating action on *i*. For every predecessor *j *of *i*, *sign*(*j *→ *i*) * *sign*(*X*_*j*_) provides the sign of the *influence *of *j *on the species *i*. Then, we can write a constraint on the variation to interpret the rule that was previously stated: *the variation of species i is explained by the variation of at least one of its predecessors in the graph*.

(1) $$sign(X_{i})\approx \sum_{j\to i}sign(j\to i)sign(X_{j}).$$

When the experiment is a genetic perturbation, the same equation holds for every node that was not genetically perturbed during the experiment and such that all its predecessors were not genetically perturbed. If a predecessor *X*_*M *_of the node was knocked-out, the equation becomes

(2) $$\begin{array}{lll} sign(X_{i}) & \approx & -sign(M\to i)\\ & & +\sum_{\begin{array}{l} j\to i\\ j\neq M \end{array}}sign(j\to i)sign(X_{j}). \end{array}$$

The same holds with +*sign*(*M *→ *i*) when the predecessor *X*_*M *_was over-expressed. There is no equation for the genetically perturbed node.

The *sign algebra *is the suitable framework for reading these equations [26]. It is defined as the set {+, -, **?**, **0**}, provided with a sign consistency relation ≈, and arithmetic operations + and ×. The following tables describe this algebra:

$$\begin{array}{c} \begin{array}{cccc} ++-=? & +++=+ & +\times -=- & +\times +=+\\ -+-=- & ++0=+ & -\times -=+ & +\times 0=0\\ 0+0=0 & -+0=- & 0\times 0=0 & -\times 0=0\\ ?+-=? & ?++=? & ?\times -=? & ?\times +=?\\ ?+?=? & ?+0=? & ?\times ?=? & ?\times 0=0 \end{array}\\ \begin{array}{cccccc} +≉- & +\approx 0 & -\approx 0 & ?\approx + & ?\approx - & ?\approx 0 \end{array} \end{array}$$

For a given interaction graph *G*, we will refer to the *qualitative system *associated with *G *as the set made up by applying constraint (1) for each node in *G*. We say that node variations *X*_*i *_∈ {+, -, **0**} are *consistent *with the graph *G *when they satisfy all the constraints associated with *G *using the sign consistency relation ≈.

With this material at hand, let us come back to our original problem, namely to infer the regulatory role of TFs from the combination of heterogeneous data. In the following we assume that:

• The interaction graph is either given by a model to be validated, or built from ChIP-chip data and TF binding site search in promoter sequences. Thus, as soon as a TF *j *binds to the promoter sequence of gene *i*, *j *is assumed to regulate *i*. This is represented by an arrow *j *→ *i *in the interaction graph.

• The regulatory role of a TF *j *on a gene *i *(as inducer or repressor) is represented by the variable *S*_*ji*_, which is constrained by Eqs. (1) or (2).

• Expression profiles provide the sign of variation of the gene expression for a set of *r *steady-state perturbation, mutant, or over-expression experiments. In the following, $x_{i}^{k}$ will stand for the sign of the observed variation of gene *i *in experiment *k*.

Our inference problem can now be stated as finding values in {+, -} for *S*_*ji*_, subject to the constraints:

(3) $$\begin{array}{l} \text{for all }(1\leq i\leq n),(1\leq k\leq r),\text{s}\text{.t}\text{. }i\;\text{is not}\\ \text{genetically perturbed in the }k\text{-th experiment}\\ \{\begin{array}{ccc} x_{i}^{k} & \approx & \begin{array}{cc} \sum_{j\to i}S_{ji}x_{j}^{k} & \text{if all predecessors }j \end{array}\\ & & \text{are not genetically perturbed}\\ x_{i}^{k} & \approx & -S_{Mi}+\sum_{j\to i,j\neq M}S_{ji}x_{j}^{k}\\ & & \text{if }M\;\text{is knocked-out}\text{.}\\ x_{i}^{k} & \approx & S_{Mi}+\sum_{j\to i,j\neq M}S_{ji}x_{j}^{k}\\ & & \text{if }M\text{ isover-expressed}. \end{array} \end{array}$$

Most of the time, this inference problem has a huge number of solutions. However, some variables *S*_*ji *_may be assigned the same value in *all *solutions of the system. Then, the recurrent value assigned to *S*_*ji *_is a logical consequence of the constraints (3), and a prediction of the model. We will refer to these inferred interaction signs as *predictions *of the qualitative system, that is, sign variables *S*_*ji *_that have the same value in all solutions of a qualitative system (3). When the inference problem has *no solution*, we say that the model and the data are *inconsistent *or *ambiguous*.

**Figure 4:**
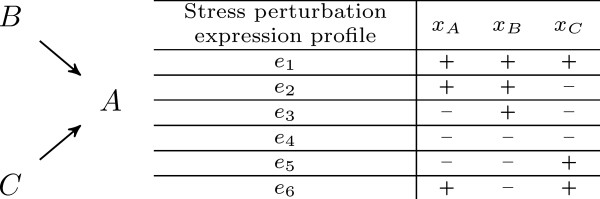
Interaction graph of three genes *A*, *B*, *C*, where their changes in expression was observed in six stress perturbation experiments.

Let us illustrate this formulation with a very simple (yet informative) example. Suppose that we have a system of three genes *A*, *B*, *C*, where *B *and *C *influence *A*, as given in Fig. 4. Let us say that for this interaction graph we obtained six experiments, and in each of them the variation of all products in the graph was observed. Using some or all of the experiments provided will lead us to different qualitative systems, as shown in Table 1, hence to different inference results.

**Table 1:** Illustration of the sign inference process

Experiments used	Qualitative system	Replacing values from experiments	Consistent solutions (*S*_*BA*_,*S*_*CA*_)	Inferred signs (identical in all solutions)
{*e*_1_}	$x_{A}^{1}$	(+) ≈ *S*_*BA *_× (+) + *S*_*CA *_× (+)	(+, +) (+, -) (-, +)	∅

{*e*_1_, *e*_2_}	$x_{A}^{1}$	(+) ≈ *S*_*BA *_× (+) + *S*_*CA *_× (+)	(+,+)	{*S*_*BA *_= +}
	$x_{A}^{2}$	(+) ≈ *S*_*BA *_× (+) + *S*_*CA *_× (-)	(+, -)	

{*e*_1_, *e*_2_, *e*_3_}	$x_{A}^{1}$	(+) ≈ *S*_*BA *_× (+) + *S*_*CA *_× (+)	(+, +)	{*S*_*BA *_= +, *S*_*CA *_= +}
	$x_{A}^{2}$	(+) ≈ *S*_*BA *_× (+) + *S*_*CA *_× (-)		
	$x_{A}^{3}$	(-) ≈ *S*_*BA *_× (+) + *S*_*CA *_× (-)		

In this example the variables are only the roles of regulations (signs) in the interaction graph. Variations of the species in the graph are obtained from six experiments. Using different sets of experiments we infer different roles of regulation. Using experiments {*e*_1_, *e*_2_, *e*_3_}, for example, our qualitative system will have three constraints and not all valuations of variables *S_BA_*and *S_CA_*satisfy this system according to the sign algebra rules. As we obtain unique values for these variables in the solution of the system, we consider them as inferred (predicted).

### Algorithmic procedure

When the signs on edges of the interaction graph are known (*i.e*. fixed values of *S*_*ji*_), finding consistent node variations *X*_*i *_is a NP-complete problem [26]. When the node variations are known (*i.e*. fixed values of *X*_*i*_), finding the signs of edges *S*_*ji *_from *X*_*i *_can be proven NP-complete in a very similar way. However, we have been able to design algorithms that perform efficiently on a wide class of regulatory networks. These algorithms predict signs of the edges when the network topology and the expression profiles are consistent. In case of inconsistency, though, they identify ambiguous motifs and propose predictions on parts of the network that are not concerned with ambiguities.

The general process flow is as follows (see the Methods section for details):

**Step 1 **Sign Inference

Divide the graph into motifs (each node with its predecessors). For each motif, find sign valuations (see Algorithm 1 in the Appendix section) that are consistent with all expression profiles. If there are no solutions, call the motif *Multiple Behaviours Module (MBM) *and remove it from the network.

Solve again the remaining equations and determine the edge signs that are fixed to the same value in all the solutions. These fixed signs are called *predicted edge *and represent our predictions.

**Step 2 **Global test/correction of the inferred signs

Solutions at the previous step are not guaranteed to be global. Indeed, two node motifs at step 1 can be consistent separately, but not altogether (with respect to all expression profiles). This step checks global consistency by solving the equations for each expression profile. New *Multiple Behaviours Modules *can be found and removed from the system.

**Step 3 **Extending the original set of observations

Once all conflicts have been removed, we get a set of solutions in which signs are assessed to both nodes and edges. *Predicted nodes*, representing inferred gene variations can be found in the same way as we did for edges. We add the new variations to the set of observations and return to step 1. The algorithm is iterated until no new signs are inferred.

**Step 4 **Filtering predictions

In the inconsistent case, the validity of the predictions depends on the accuracy of the model and on the correct identification of the MBMs. The model can be incomplete (missing interactions), and MBMs are not always identifiable in a unique way. Thus, it is useful to sort predictions according to their reliability. Our filtering parameter is a positive integer *k *representing the number of different experiments with which the predicted sign is consistent. For a filtering value *k*, all the predictions that are consistent with less than *k *profiles are rejected.

**Figure 5:**
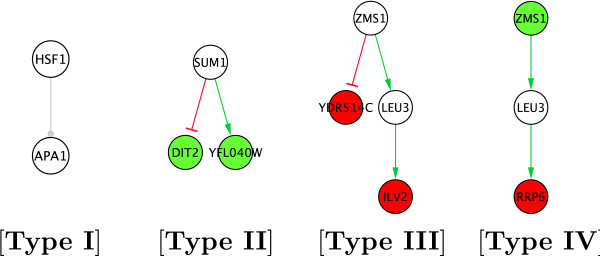
**Classification of the Multiple Behaviours Modules (MBM) found in *S. cerevisiae *transcriptional network. **Green and red interactions correspond to inferred activations and repressions respectively. Significant differentially expressed genes of the MBM, during one experimental condition, are coloured green (up-regulated), or red (down-regulated) (a) **Type I **modules are composed by regulations towards the same gene. Regulations in this module were found to be inconsistent in at least 2 experiments. (b) **Type II **are composed by genes regulated by the same direct predecessor. Explanation: The interaction among Sum1 and *YFL040W *is inferred at the Step 1 of the algorithm as an activation, while among Sum1 and *DIT2 *as an inhibition. During the *correction *step (Step 2), expression profiles related to one experiment showed that the expression of these two genes (*YFL040W *and *DIT2*) is up-regulated. As it is impossible to state if *SUM1 *is up or down-regulated (inconsistency), we mark this module as MBM. (c) **Type III **are composed by coloured genes that share a predecessor. (d) **Type IV **are composed by coloured genes sharing the same predecessor or successor.

The inference process then generates three results:

1. *A set of MBMs*, containing interactions whose role was unclear and generated inconsistencies. We have identified several types of MBMs:

• Modules of Type I: are composed of several direct regulations towards the same gene. They are detected in the Step 1 of the algorithm, and most of them are composed of only one edge like illustrated in Fig. 5, but bigger examples exist.

• Modules of Type II, III, IV: are detected in Steps 2 or 3, hence they contain either direct regulations coming from the same protein or indirect regulations and/or loops. Each of these regulations represents a consensus of all the experiments, but when we attempt to assess them globally, they lead to contradictions. The indices II-IV have no topological meaning, they label the most frequent situations and are illustrated in Fig. 5.

2. *A set of inferred signs*, meaning that the expression profiles fix the signs of certain interactions in a unique way.

3. *A reliability ranking of inferred signs*. The filtering parameter *k *used for ranking is the number of different expression profiles that validate a given sign.

On a computational level, the division between Step 1 (which considers each small motif with all profiles together) and Step 2 (which considers the whole network with each profile separately) is necessary to overcome the memory complexity of the search for solutions. To handle large scale systems we combine decision diagrams and constraint solvers (see details in the Methods section).

Since our basic rule is a weak constraint, we expect it to produce very robust predictions. On the other hand, there are theoretical limits to this approach. For certain interaction graphs, not a single sign may be inferred even with a high number of experiments. In the next paragraphs, we comment on the maximum number of signs that can be inferred from a given graph.

In perturbation experiments, gene responses are observed following changes of external conditions (temperature, nutritional stress, *etc*.), gene inactivations, knock-outs, or over-expression. When one expression profile is available for all the genes in the network we say that we have a *complete profile*, otherwise the profile is *partial *(data is missing).

In the following pragraphs we describe the results we obtained. First of all, in order to validate our formal approach, we evaluated the percentage of the *E. coli *network recovered from a reasonable number of artificial randomly generated perturbation experiments. Secondly, we combined real perturbation experiments with the *E. coli *network and computed the percentage of the recovered network. Finally, we performed the same previous analysis in a real setting of the *S. cerevisiae *network obtained from ChIP-chip data.

On a computational level, we checked that our algorithms were able to handle large scale data, as produced by high-throughput measurement techniques (expression arrays, ChIP-chip data). This is demonstrated in the following by considering networks of thousands of genes.

### Stress perturbation experiments: how many do you need?

For any given network topology, even when considering all possible experimental profiles, there are signs that cannot be determined (see Table 1). Sign inference has thus a theoretical limit, referred to here as *theoretical percentage of recovered signs*, that is unique for a given network topology. If only some perturbation experiments are available, and/or data is missing, the percentage of inferred signs will be lower. For a given number *N *of available expression profiles, *the average percentage of recovered signs *is defined over all sets of *N *different expression profiles consistent with the qualitative constraints Eqs. (1) and (2).

In order to calculate the theoretical and the average percentages of recovered signs for the transcriptional network of *E. coli*, we modelled the network as an interaction graph using the public database RegulonDB [6]. For each transcriptional regulation *A *→ *B *we added the corresponding arrow between genes *A *and *B *in the interaction graph. This graph will be referred to as the *unsigned interaction graph*.

From the unsigned interaction graph of *E. coli*, we build the *signed interaction graph *by annotating the edges with a sign. Most of the time, the regulatory role of a TF is available in RegulonDB, however, when it is unknown or depends on the TF level, we arbitrarily choose the value + for this regulation. This provides a graph with 1529 nodes and 3802 edges, all signed edges. The signed interaction graph is used to generate *complete expression profiles *that simulate the effect of perturbations. More precisely, a perturbation experiment is represented by a set of gene expression variations {*X*_*i*_}_*i *= 1,...,*n *_that are not entirely random, for they are constrained by Eqs.(1) and (2). Then, we forget the signs of the network edges and compute the qualitative system with the signs of regulations as unknown.

The *theoretical maximum percentage of inference *is given by the number of signs that can be recovered assuming that *complete *expression profiles of *all *conceivable perturbation experiments are available. We computed this maximum percentage using constraint solvers (see Algorithm 2 in the Appendix section). We found that *at most *40.8% of the signs in the network can be inferred, corresponding to *M*_*max *_= 1551 edges.

**Figure 6:**
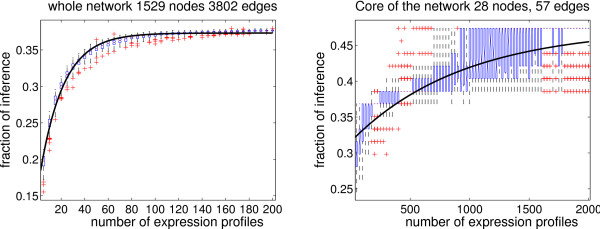
**(Both) Statistics of the sign-inference process on the regulatory network of *E. coli *from *complete *expression profiles.** The signed interaction graph is used to generate sets of *N *random artificial expression profiles which cover the *whole *network. Then, each set of *N *profiles is used with the unsigned interaction graph to recover regulatory roles. X-axis: number *N *of expression profiles in the dataset. Y-axis: percentage of recovered signs in the unsigned interaction graph. Each set of *N *random profiles was generated 100 times; the distribution of the recovered signs is plotted as a boxplot. The continuous line corresponds to the theoretical formula *Y *= *M*_1 _+ *M*_2_(1 - (1 - *p*)^*X*^); *M*_1 _denotes the number of single incoming regulations inferred with probability one from any complete profile (using the naive inference algorithm), and *M*_2 _denotes the number of signs inferred with a probability *p *(0 <*p *< 1) per experiment. (Left) Statistics using the *whole E. coli *regulatory network. We estimated that at most 37.3% of the network can be inferred from a small number of different complete profiles. Among the inferred regulations, we estimated to *M*_1 _= 609 the number of signs inferred with probability one from any complete expression profile. The remaining *M*_2 _= 811 signs are inferred with a probability whose average is *p *= 0.049 per experiment. Hence, 30 perturbation experiments are enough to infer 33% of the network. (Right) Statistics using only the *core *of the former graph (see definition of a core in the text). We estimated *M*_1 _= 18 and *M*_2 _= 9, implying that the maximum rate of inference is 47.4%. Since *p *= 0.0011, the number of expression profiles required to obtain a given percentage of inference is greater than in the case using the whole network (*N *= 100 to infer 33% of the network).

However, this maximum can be obtained only if all conceivable (more than 2^50^) perturbation experiments are done, which is in practice not possible. We performed computations to understand the influence of the number of experiments (*N*) on the inference. For each value of *N *(from 5 to 200), we generated 100 sets of *N complete *random expression profiles and performed our algorithm for each set. Then, the percentage of inference was calculated as a function of *N*. The resulting statistics are shown in Fig. 6.

We can obtain a theoretical formula explaining the saturation aspect of the curve in Fig. 6. Let us suppose that the network contains *M*_1 _single incoming regulations. These can be inferred with probability one from only one experiment, using the naive algorithm (see Algorithm 1). Let us suppose a second category of interactions, whose signs are inferred with probability *p *(0 <*p *< 1) on average, per experiment. This implies that the average number of inferred signs for one experiment is *M*(1) = *M*_1 _+ *pM*_2_, where *M*_2 _is the number of interactions in the second category. Supposing now that inference failures are independent for different experiments, we obtain the average number of inferred signs for *N *experiments: *M*(*N*) = *M*_1 _+ *M*_2_(1 - (1 - *p*)^*N*^). In general, we have *M*_1 _+ *M*_2 _<*E *(*E *is the total number of edges), meaning that there are edges whose signs cannot be inferred.

In our example, the value *M*_1 _= 609 corresponds to the average number of signs inferred by the naive algorithm. Surprisingly, by using our method we can significantly improve the naive inference with little effort. For the whole *E. coli *network it appears that a few expression profiles are enough to infer a significant percentage of the network. More precisely, 30 different expression profiles may be enough to infer one third of the network (1267 regulatory roles). Adding more expression profiles continuously increases the percentage of inferred signs. For *N *> 100 we are practically on the plateau close to 37.3% (this corresponds to *M *= 1420 signed regulations).

According to our estimates the position of the plateau is *M *= *M*_1 _+ *M*_2 _= 1420, which is smaller than the theoretical maximum *M *<*M*_*max*_. The difference, although negligible in practice (to obtain *M*_*max *_one has to perform *N *> 2^50 ^experiments), suggests that the plateau has a very weak slope. This means that contributions of different experiments to sign inference are weakly dependent.

The values of *M*_1_*, M*_2_, *p *estimate the efficiency of our method: large *p*,*M*_1_,*M*_2 _mean small number of expression profiles needed for inference.

### Inferring the core of the network

**Figure 7:**
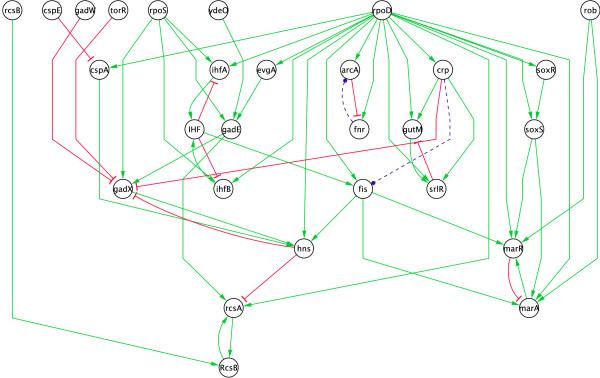
**Core of *E. coli *network.** It consists of all oriented loops and of all oriented chains leading to loops. The core contains the dynamical information of the network, hence sign edges are more difficult to infer.

Obviously, not all interactions play the same role in the network. The *core *is a subnetwork that naturally appears for computational purposes and plays an important role in the system. It consists of all oriented loops and of all oriented chains leading to loops. All oriented chains leaving the core without returning are discarded when reducing the network to its core. Acyclic graphs and in particular trees have no core. The main property of the core is that if a system of qualitative equations has no solution, neither has the reduced system built from its core. Hence it corresponds to the most difficult part of the constraints to solve. It is obtained by reduction techniques that are very similar to those used in [28] (see details in the Methods section). As an example, the core of *E. coli *network (shown in Fig. 7) only has 28 nodes and 57 edges.

In the previous section, we applied the same inference process to this graph. Not surprisingly, we noticed a rather different behaviour when inferring signs on a core graph than on a whole graph as demonstrated in Fig. 6. In the former case, we needed many more experiments for the inference since the sets of expression profiles contained from *N *= 50 to 2000 random profiles.

Two observations may be concluded. First, a greater number of experiments is required to reach a comparable percentage of inference; the value of *p *is smaller than for the whole network. This confirms that the core is more difficult to infer than the rest of the network. Second, Fig. 6 displays a much less continuous behaviour for the core. More precisely, when using the core, different perturbation experiments have a strongly variable impact on sign inference. For instance, the experimental maximum percentage of inference (27 signs over 58) can be obtained already from about 400 expression profiles, yet, most of the datasets with 400 profiles infer only 22 signs.

This suggests that not only the core of the network is more difficult to infer, but also that a brute force approach (multiplying the number of experiments) may fail as well. This situation encourages us to apply experiment design and planning, that is, computational methods to minimise the number of perturbation experiments while inferring a maximal number of regulatory roles.

This also illustrates why our approach is complementary to dynamical modelling. In the case of large scale networks, when an interaction stands outside the core of the graph, an inference approach is suitable for inferring the sign of the interaction. However, when an interaction belongs to the core of the network, more complex behaviours occur (*e.g*. influences that depend on activation thresholds) thus, a precise modelling of the dynamical behaviour of this part of the network should be performed [29].

### Influence of missing data

In the previous paragraphs, we assumed that all products in the network were observed. That is, in each experiment each node is assigned a value in {+, **0**, -}. However, in real measurement devices, such as expression profiles, a part of the values is discarded due to technical reasons. A practical method for network inference should cope with missing data.

**Figure 8:**
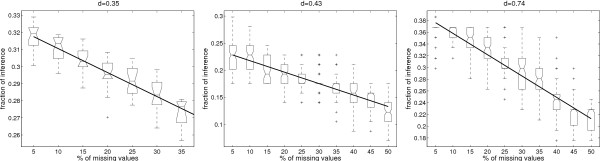
**(All) Statistics of the sign-inference process on the regulatory network of *E. coli *from *partial *expression profiles.** The setting is similar to the one used in Fig. 6, except for the cardinal of the expression profiles (*N *is fixed), and for the variable on X-axis which represents the percentage of missing values in the expression profiles. The continuous line corresponds to the theoretical prediction *M*_*i *_= $M_{i}^{max}$ - d * f * *M*_*total*_; where $M_{i}^{max}$ is the number of inferred interactions from *complete *expression profiles, *d *is the number of interaction signs no longer inferred when a node is not observed, *f *is the fraction of unobserved nodes, and *M*_*total *_is the total number of nodes. (Left) Statistics for the whole network; we used 30 sets of artificial expression profiles (*N *= 30). We estimated *d *= 0.35, meaning that on average we lose one interaction sign for about 2.9 missing values in the profiles. (Middle) Statistics for the core network (*N *= 30). We estimated *d *= 0.43; the core of the network, however, is more sensitive to missing data. (Right) Statistics for the core network (*N *= 200). We estimated *d *= 0.74; hence, increasing the number of expression profiles increases the sensitivity to missing data.

We studied the impact of missing values on the percentage of inference. For this, we have considered a fixed number of expression profiles (*N *= 30 for the whole *E. coli *network, *N *= 30 and *N *= 200 for its core). Then, we have randomly discarded a growing percentage of observed products in the profiles, and computed the percentage of inferred regulations. The resulting statistics are shown in Fig. 8.

In both cases (whole network and core), the dependency between the average percentage of inference and the percentage of missing values is qualitatively linear. Simple arguments allow us to find an analytic dependency. If not observing one node of the network implies losing information on *d *interaction signs, we are able to obtain the following linear dependency *M*_*i *_= $M_{i}^{max}$ - d * f * *M*_*total*_; where $M_{i}^{max}$ is the number of inferred interactions for complete expression profiles (no missing values), *f *is the fraction of unobserved nodes, and *M*_*total *_is the total number of nodes. In order to keep *M*_*i *_non negative, *d *must decrease with *f*. Our numerical results imply that the constancy of *d *and the linearity of the above dependency extend to rather large values of *f*. This indicates that our qualitative inference method is robust enough for practical use. For the whole network we estimated *d *= 0.35, meaning that on average we lose one interaction sign for about 2.9 missing values. However, for the same number of expression profiles, the core of the network is more sensitive to missing data (the value of *d *is larger, it corresponds to losing one sign for about 2.3 missing values). For the core, increasing the number of expression profiles increases *d *and hence the sensitivity to missing data.

### Application to *E. coli *network with a real compendium of expression profiles

We validated our method on the transcriptional *E. coli *network using the compendium of expression profiles publicly available in [9] and [22]. This time the network was composed of 1418 nodes and 2888 edges. The difference with the previous model are the sigma-factors – gene interactions.

Several profiles were available, including a reference condition. We grouped together the different profiles corresponding to the same experiment; for each gene we calculated its average variation in the group of profiles. When profiles were time series, we considered that each time series ends with steady state and we used the last state in the time series. Then, we sorted the measured genes in four classes: 2-fold up-regulated, 2-fold down-regulated, non-observed, and zero variation; this last class corresponds to non significantly (2-fold) expressed genes. Only the first two classes were used in the algorithm. Therefore, there will be missing data: for some edges, neither the input nor the output are observed. Altogether, we have processed 226 sets of expression profiles corresponding to 61 different experiments (over-expression, gene-deletion, and stress perturbation). We verified, for all the experiments, that they correspond to the comparison between one perturbed condition against a control condition with identical levels in all chemical components except for the one altered in the perturbed condition.

We applied our inference algorithm twice: the first time we used the signed network in a pre-processing step, in order to clean the expression data. It appears that the signed network is consistent with only 31 of the 61 selected experiments. After discarding the inconsistent motifs from each experiment (deleting observations that caused conflicts), we stayed with 61 experiments which only contained the data consistent with the signed network. In these 61 experiments, on average 12.62% of the network nodes were observed. When summing up all the observations, we obtained that 6.5% (190) of the edges (input and output) were observed in at least one expression profile; these represent the maximal set of signs that can be inferred at Steps 1 and 2 of our inference algorithm. In order to test our algorithm we wiped out the information on edge signs and then tried to recover it. Since the profiles and network were consistent, our algorithm found no ambiguity and predicted 38 signs, *i.e*. 20% of the edges observed at least once (input and output). The naive inference algorithm inferred 31 signs. Hence, 18% of the total of our predictions could not be obtained by the naive algorithm.

**Figure 9:**
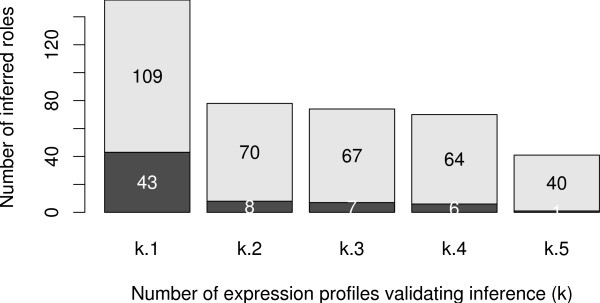
**Results of the inference algorithm applied to *E. coli *network with a compendium of 61 experiments not globally coherent. **The dark and light regions of the bars correspond to false positive and validated predictions, respectively. Without filtering, there are 28.3% of false positives. With filtering – keeping only the sign predictions confirmed by *k *different experiments – the rate of false positives decreases to 2.5%.

Afterwards, we tested our algorithm with the full set of observations, no data being discarded. Conflicts appeared and we filtered our inference with different parameters on the full set of 61 experiments including inconsistencies. This time 12.9% of the network products were observed on average. When summing all the observations, 17.2% (497) of the edges (input and output) were observed in at least one expression profile. Several values of the filtering parameter *k *were used from *k *= 1 to *k *= 5. Without filtering we predicted 152 signs of the network (30% of the edges observed at least once), among them, 41.4% were not inferred by the naive algorithm. We compared the predictions to the known interaction signs: 28.3% of the predictions were false predictions. Sources of errors may lie on non-modelled interactions (possibly effects of sigma-factors), or in using experiments on different *E. coli *strains. Filtering improves our score allowing us to retain only reliable predictions. Thus, for *k *= 5, we inferred 41 signs, of them, only 1 was an incorrect prediction (2.5% of false prediction). We conclude that filtering is a good way to strengthen our predictions even when the model is not precise enough. We illustrated the effect of the filtering process in Fig. 9.

It should be noted that we obtained very similar results either by cleaning the data thanks to the signed network, either by using our filtering procedure. This is a particularly clear indication that this filtering procedure is an effective strategy to produce robust predictions.

**Figure 10:**
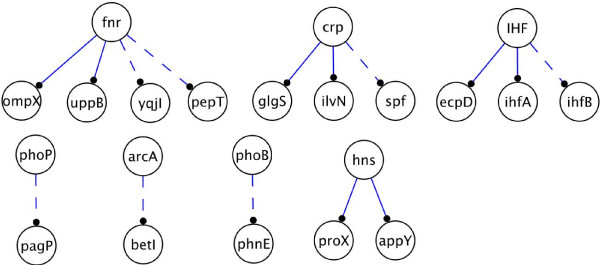
**Interactions in the regulatory network of *E. coli *that are ambiguous with a compendium data of expression profiles.** For each interaction, there exist at least two expression profiles that do not predict the same sign on the interaction. Dotted and filled lines represent the MBM of Type I and Type II, respectively.

Our algorithm also detected ambiguous modules in the network. There are seven MBM of Type I (*i.e*. single incoming interactions); four of them are also stated as ambiguous by the naive algorithm. In addition, there are 4 MBM of Type II that are not detected by the naive inference algorithm. All the ambiguities are shown in Fig. 10. A list of experimental assays that yield ambiguities on each interaction is given in the Supplementary Web site. This analysis shows that there exist non-modelled interactions that balance the effects on the targets in the MBM detected.

### A real case: inference of signs in *S. cerevisiae *transcriptional regulatory network

We applied our inference algorithm to the transcriptional regulatory network of the budding yeast *S. cerevisiae*. Let us here briefly review the available sources that can be used to build the unsigned regulatory network. The experimental dataset proposed by Lee *et al*. [11] is widely used in the network reconstruction literature. It is a study conducted under nutrient rich conditions, and it consists of an extensive ChIP-chip screening of 106 TFs. Estimations regarding the number of yeast TFs that are likely to regulate specific groups of genes by direct binding to the DNA vary from 141 to 209, depending on the selection criteria. In follow-up papers of this work, the ChIP-chip analysis was extended to 203 yeast TFs in rich media conditions and 84 of these regulators in at least one environmental perturbation [12]. Analysis methods were refined in 2005 by MacIsaac *et al*. [13]. Other studies continued to work in this network using different approaches [10,14-16]. Here we selected two of these sources. All networks are provided in the Supplementary Web site.

(A) The first network consists of the core of the transcriptional ChIP-chip regulatory network produced in [11]. Starting from the full network with a p-value of 0.005, we reduced it to the set of nodes that have at least one output edge. This network was already studied in [28]. It contains 31 nodes and 52 interactions.

(B) The second network contains all the transcriptional interactions between TFs shown by [11] with a p-value below 0.001. It contains 70 nodes and 96 interactions.

(C) The third network is the set of interactions among TFs as inferred in [13] from sequence comparisons. We have considered the network corresponding to a p-value of 0.001 and 2 bindings (83 nodes, 131 interactions).

(D) The last network contains all the transcriptional interactions among genes and regulators shown by [11] with a p-value below 0.001. It contains 2419 nodes and 4344 interactions.

#### Inference process with gene-deletion expression profiles

We first applied our inference algorithm to the large scale network (D) using a panel of expression profiles for 210 gene-deletion experiments [30]. The information given by this panel is quite small, since 1.6% of all the products in the network is on average observed, and 12% of the edges (input and output) of the network are observed in at least one expression profile. Using these data, we inferred 162 regulatory roles.

We validated our prediction with a literature-curated network on Yeast [31]. We found that among the 162 sign-predictions, 12 were referenced with a known interaction in the database, and 9 with a good sign.

Gene-deletion expression profiles were used in order to compare our results to path analysis methods [20,23] since the latter can only be applied to knock-out data. Other sign-regulation inference methods needed either other sources of gene-regulatory information (promoter binding information, protein-protein information), or time-series data to be performed [10,15,18].

First, we tested the consistency between the inferred network obtained from path analysis methods with the 210 gene-deletion experiments. We obtained that the network was inconsistent with 28 of the 210 experiments. Second, we compared the inference results for both methods, our approach and the path analysis method, obtaining in the latter that 234 roles of widely connected paths were inferred; whereas with our method 162 roles were inferred, mainly localised in the branches of the network. Both results intersected on 17 interactions and no contradiction in the inferred role was reported. An illustration of these results is given in the Supplementary Web site.

This suggests that our approach is complementary to path analysis methods. Our explanation is as follows: in [20,23], network inference algorithms identify probable paths of physical interactions connecting a gene knock-out to genes that are differentially expressed as a result of that knock-out. This leads to a search for the smallest number of interactions that carry the largest information in the network. Hence, inferred interactions are located near the core of the network, but not exactly in the core. On the contrary, as we already mentioned, the combinatorics of interactions in the core of the network are too intricate to be determined from a few hundreds of expression profiles with our algorithm, thus, we concentrate on interactions around the core.

#### Inference with stress perturbation expression profiles

To overcome the problem exposed using the small amount of information contained in [30], we have used stress perturbation experiments. These data correspond to curated information available in SGD (Saccharomyces Genome Database) [32]. When time series profiles were available, we selected the last time expression array. Therefore, we collected and treated 15 experiments described in Table 2. For each expression array, we sorted the measured genes in four classes: 2-fold up-regulated, 2-fold down-regulated, non-observed, and zero variation. Full datasets are available in the Supplementary Web site.

**Table 2:** List of genome expression experiments on *S. cerevisiae *used in the sign inference process

Experiment Identifier	Description	Ref.
E1	Diauxic Shift	[40]
E2	Sporulation	[41]
E3	Expression analysis of Snf2 mutant	[42]
E4	Expression analysis of Swi1 mutant	[42]
E5	Pho metabolism	[43]
E6	Nitrogen Depletion	[44]
E7	Stationary Phase	[44]
E8	Heat Shock from 21°C to 37°C	[44]
E9	Heat Shock from 17°C to 37°C	[44]
E10	Wild type response to DNA-damaging agents	[45]
E11	Mec1 mutant response to DNA-damaging agents	[45]
E12	Glycosylation defects on gene expression	[46]
E13	Cells grown to early log-phase in YPE (Rich medium with 2% of Ethanol)	[47]
E14	Cells grown to early log-phase in YPG (Rich medium with 2% of Glycerol)	[47]
E15	Titratable promoter alleles – Ero1 mutant	[48]

All experiments contain information on steady state shift and their curated data is available in SGD (Saccharomyces Genome Database) [32].

As in the case of *E. coli*, it appeared that all the networks (A), (B), (C), and (D) were not consistent with the whole set of expression arrays. Thus, when executing our algorithms we identified motifs that held ambiguities, and we marked them as MBM of type I-IV (as described in our inference algorithm). We also generated a set of inferred signs and applied the filtered algorithm (with filter *k *= 3) to the large scale network (D).

We obtained our total inference rate by adding the number of inferred signs fixed in an unique way to the number of non-repeated interactions in the MBM detected, and dividing it by the total number of edges in the network. In Table 3 we illustrate the inference rate obtained for each of the networks. Depending on the network, the inference rate varies from 19% to 37%; thus, they are similar to the theoretical rates obtained for *E. coli *network even with a small number of perturbation experiments (14 or 15).

**Table 3:** Results of the sign inference process on *S. cerevisiae*

Interaction network	Nodes	Edges	Average observed nodes	In/Out observed simultan.	Inferred signs {+, -}	MBM Type I	MBM Type II-IV	Total Inference	Naive Algorithm Inference
(A) Core of Transc. Network [11,28]	31	52	28%	88%	11	3	0	26.8%	11%
(B) Extended Transc. Network [11]	70	96	26%	72%	29	7	0	37.4%	15,6%
(C) MacIsaac inferred network [12,13]	83	131	33%	69%	21	4	0	19%	11%
(D) Global Transc. Network [11]	2419	4344	30%	52%	no filter : 631 filter k = 3 : 198	281	463	32%	13.9%

Sign inference process applied on four transcriptional networks of *S. cerevisiae*. 15 significant (2-fold) experiments were used for the inference. The *In/Out observed simultaneously *rate refers to the possible sign inference rate if all observations of the in/out nodes of one edge lead to predictions. The *Inferred signs *are the number of signs fixed in an unique way {+, -} by all the experiments; *MBM Type I*, refers to the number of edges found in a module of this type; and analogusly for *MBM Type II-IV*. The sum of these 3 values divided by the total number of edges will represent the rate of the *Total Inference*. In addition, we calculated the inference rate obtained with the Naive Algorithm. For network (D) all the inference rates were obtained using the algorithm without filtering, except for the number of *Inferred signs*.

**Figure 11:**
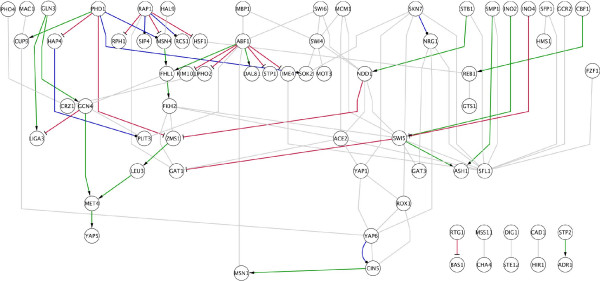
***S. cerevisiae***** transcriptional network**. Only interactions among transcription factors were taken into account (70 nodes, 96 edges) [11]. A total of 29 interactions were inferred. Green and red arrows correspond to inferred activations and repressions, respectively. Blue arrows correspond to the detected MBM of Type I. The diagram layout was produced using the Cytoscape package [39].

We validated the inferred interactions comparing them to the literature-curated network published in [31]. We obtained 631 predictions when no filtering is applied. Furthermore, among the 198 interactions predicted with a filter parameter *k *= 3, 19 were referenced with a known interaction in the database, and only 1 prediction had a wrong sign. As in the case of *E. coli*, we conclude that filtering is a good way to produce extremely robust predictions. Additionally, we compared our predictions to the naive inference algorithm finding that the naive algorithm usually predicts half of the signs that we obtain. In Fig. 11 we illustrate the inferred interactions for Network (B).

As already mentioned, the algorithm identified a large number of ambiguities. The exhaustive list of MBM is given in the Supplementary Web site and the Type I modules of size 2 found for the networks (A), (B), and (C) are detailed in Table 4. We noticed that the MBM of Type I were detected in the four networks; whereas the MBM of Type II-IV were only detected, in an large number, for Network (D); Type II MBM being the most numerous (85.4%). For each MBM, a precise biological study of the species should enable to understand the origin of the ambiguity: erroneous expression data, missing interactions in the model, or context-dependent regulations.

**Table 4:** Ambiguous modules of Type I found for 3 transcriptional networks of *S. cerevisiae*.

Interaction network	Actor	Target	Experiment 1	Experiment 2
(A) Core of Transc. Network	YAP6	CIN5	Expression during Sporulation [41]	YPD Broth to Stationary Phase [44]
	GRF10	MBP1	YPD Broth to Stationary Phase [44]	Mec1 mutant + Heat [45]
	PDH1	MSN4	Nitrogen Depletion [44]	Heat shock 21°C to 37°C [44]
(B) Extended Transc. Network	YAP6	CIN5	Expression during Sporulation [41]	YPD Broth to Stationary Phase [44]
	RAP1	SIP4	Expression during Sporulation [41]	Expression during the diauxic shift [40]
	SKN7	NRG1	YPD Broth to Stationary Phase [44]	Expression during the diauxic shift [40]
	PHD1	SOK2	Heat shock 21°C to 37°C [44]	YPD Broth to Stationary Phase [44]
	RAP1	RCS1	Wild type + Heat [45]	Transition from fermentative to glycerol- based respiratory growth [47]
	PHD1	MSN4	Nitrogen Depletion [44]	Heat shock 21°C to 37°C [44]
	HAP4	PUT3	Expression during the diauxic shift [40]	Snf2 mutant, YPD [42]
(C) MacIssac inferred network	SWI5	ASH1	Expression regulated by the PHO path- way [43]	YPD Broth to Stationary Phase [44]
	SKN7	NRG1	YPD Broth to Stationary Phase [44]	Nitrogen Depletion [44]
	NRG1	YAP7	Expression regulated by the PHO path- way [43]	Transition from fermentative to glycerol- based respiratory growth [47]
	NRG1	GAT3	Glycosylation [46]	Transition from fermentative to glycerol- based respiratory growth [47]

For each ambiguous module, we list two inconsistent experiments that infer a different role of regulation.

### Contribution of expression profiles to the inference

Analysing only the sign inference process on the global network (D), we wish to estimate how the 14 experiments used influence the unique way {+, -} inferred signs. On that account we address the following question: Assuming that all the inferred roles in Step 1 of our inference algorithm are correct, which is the experiment that marks more inferred roles as inconsistent (*i.e*. that generates more MBM)?

**Figure 12:**
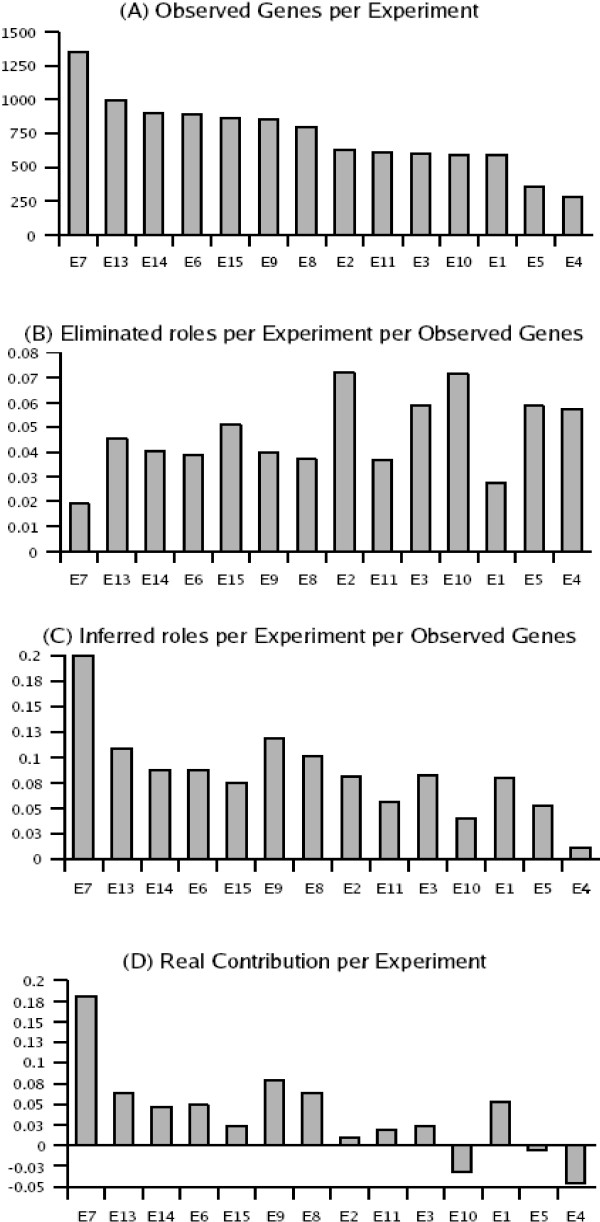
**Classification of the 14 experiments used in the sign-inference process for the global transcriptional network (2419 nodes, 4344 edges).** The experiments are represented by their identifier (see Table 2). Each experiment has a twofold contribution: it spots inconsistent modules (MBM that are further excluded from inference) and it predicts interaction roles. Some experiments have more predictive power, just because they include more genes. In order to normalise the predictive power, we divided the percentage of predictions by the percentage of observed nodes. For each experiment we have estimated: (A) Number of significant (2-fold) up/down-regulated genes. (B) Percentage of edges in the spotted MBMs of type II-IV divided by the percentage of observed genes. (C) Percentage of inferred signs divided by the percentage of observed genes. (D) Real contribution of each experiment, calculated by subtracting C (inference) from B (eliminated inconsistency); negative values correspond to experiments whose main role is to spot ambiguities.

Therefore, we classified the 14 experiments according to the MBM of Type II-IV generated per experiment. MBM of Type I are not included in this computation, for they are inferred in Step 1 of the algorithm. The results of this classification are shown in Fig. 12. The fourth chart illustrates that the real contribution of each expression profile does not depend on the amount of observed genes it contains.

## Discussion

### Predicting from a "small" number of expression profiles

In principle, inferring the functional effect of regulations could be done using general reconstruction methods. The most outstanding approaches in this domain include Bayesian networks [33], linear ordinary differential equations (ODE) [34,35] and correlation/causal networks [14,16,36] (see [10] for a review, and a comparison on several datasets). These are quantitative methods which are carefully designed to cope with the high level of noise that is generally observed in expression data. They rely either on an explicit parametric modelling of noise distribution (like in Bayesian networks), either on robust statistical estimators for the network and its kinetic parameters. The main limitation of these approaches is the number of independent samples they require in order to be properly used. It is often stated [10,36] that a minimum of 100 to 300 expression profiles are needed for the estimation procedure. While there exists a couple of datasets of such size, the usual number of available profiles for a given biological system is much smaller. Our approach is meant to be used when the number of profiles ranges from 1 to a couple of hundreds, and should thus be seen as complementary to quantitative methods. Indeed our simulations on *E. coli *network show that one can characterise about 30% of the regulations from 30 expression profiles. We additionally showed that this is close to the theoretical limit of our approach. This result was confirmed using expression data on the same network: we infer 20% of the regulations whose input and output are simultaneously observed in at least one experiment, using 61 expression profiles.

### Generating accurate predictions

The problem of inferring functional effect of transcription factors was specifically addressed by Yeang and colleagues [20,23], using a probabilistic discrete model. In this approach, one identifies probable paths of physical interactions connecting a gene knock-out to genes that are differentially expressed as a result of that knock-out. Predictions correspond to the signs found in models of maximum likelihood. More generally, most reconstruction methods are based on computing an "optimal" model with respect to the data. This raises two main issues. First, the underlying optimization problems are often non convex, and finding a global optimum is a very difficult computational task. In practice, most algorithms only guarantee to find a local optimum, which should be cautiously examined before being reported as a prediction. Second, even if a global optimum is found, it is important (but computationally difficult) to check that there is no slightly sub-optimal model that yields very different predictions. In other terms, it is necessary to evaluate the *robustness *of the predictions. In our approach, we describe the (possibly huge) set of models that are consistent with the data, then look for invariants in this set. This means that our predictions are compatible with *all *feasible models. In order to cope with experimental noise, we combine this strategy with a filtering procedure, which selects predictions that agree with a minimal number of expression profiles. This led us to very accurate predictions, as it was shown on data from *E. coli *and yeast. We compared our inference approach to the path analysis method by Yeang and colleagues [20,23]. We found that both algorithms infer a similar number of regulations, and that the predictions coincide. We noticed that the predictions are located in different parts of the network, depending on the algorithm: path analysis tends to infer signs in highly connected regions, while our approach infer signs on regulations acting on small in-degree nodes. Another difference is that path analysis requires expression profiles from gene-deletion experiments, whereas our method gives better results with stress perturbation experiments (though it can be applied to both types of experiment).

### Sign inference and network topology

Using simulations, we evaluated the dependence between the number of available expression profiles and the number of signs that can be inferred from them. Not surprisingly, we noticed that the topology of the regulatory network has a strong influence on the estimated relationship. This was illustrated by computing statistics on both a complete regulatory network and its core. The complete network is characterised by an over-representation of feedback-free regulatory cascades, which are controlled by a small number of TFs. In this setting, the number of inferred signs grows almost continuously with the number of observations. In contrast, the core network does not obey the simple law "the more you observe, the better", some expression profiles being clearly more informative than others. Additionally, in these core networks an unfeasible number of experiments is necessary to infer a small number of signs with high probability. For these core networks, two different strategies may be adopted. First, to build a more accurate model for these restricted subnetworks using dynamic modelling techniques (see [29] for a review). Second, to develop experiment planning in our qualitative framework: given some control parameters, how to find the most informative experiments while keeping their number as low as possible?

## Conclusion

In this work we proposed a discrete approach for a particular case of reconstruction problem: given a set of regulations between genes, and a set of expression profiles, determine the functional effect of each regulation, as activation or inhibition. Our approach is based on a qualitative modelling framework, that was initially introduced to check the consistency between a regulatory network and expression data [24,25]. This framework is based on a rule, which basically says that if the expression of a gene varies between two conditions, then this should be accounted for by the variation of at least one of its predecessors. Here we applied this approach to predict the functional effect of transcription factors on their target genes.

While intuitive and simple, the qualitative rule we propose can be used to infer a significant number of regulatory effects from a reasonable number of expression profiles. As shown using data on *E. coli *and yeast, the predictions are particularly reliable, especially when they are validated with our filtering procedure. Furthermore, our algorithms can handle datasets of realistic size. It should be noted that computing the predictions presented in this work requires to solve thousands of NP-hard problems (more precisely, constraints with variables on a finite domain). Each of these problem has several thousands of variables. Nevertheless, our algorithms are exact and compute the predictions in no more than an hour using a standard desktop PC. This means that they are able to cope with system-wide data in a fairly reasonable amount of time. Due to the structure of the algorithms, we are confident that they can handle even larger datasets in less time, by distributing the computations on several machines.

From our results on yeast, it appears that a significant proportion of the network – as given by ChIP-chip data – is not compatible with the available expression profiles. As explained in the Results section, these data is discarded from the analysis, in order to compute safe predictions – but at the expense of a loss of information. The subject of our current work is to develop an improved notion of prediction, that copes better with inconsistent network and data. The goal is to include inconsistent data in the inference process, while preserving the reliability of the predictions.

## Methods

### Problem statement

We consider the set of equations derived from a given interaction graph *G*:

(4) $$X_{i}^{k}\approx \sum_{j\to i}S_{ji}X_{j}^{k}\text{ for}\;1\leq i\leq n,1\leq k\leq r$$

where $X_{i}^{k}$ stands for the sign of the variation of species *i *in experiment *k*, and *S*_*ji *_the sign of the influence of species *j *on species *i*. Recall that the graph *G *itself comes from chIP-chip experiments or sequence analysis. Using expression arrays, we obtain an experimental value for some variables $X_{i}^{k}$, which will be denoted $x_{i}^{k}$; more generally uppercase (resp. lowercase) letters will stand for variables of the systems (resp. constants +, - or **0**).

A single equation in the system (4) can be viewed as a predicate *P*_*i,k*_(*X*, *S*) where *i *denotes a node in the graph and *k *one of the *r *available experiments. If the value for some variables in the equation is known, the predicate resulting from their instantiation will be denoted *P*_*i, k*_(*X*, *S*) [*x*^*k*^, *s*].

Our problem can now be stated as follows: given a set of expression profiles *x*^1^,...,*x*^*r*^, decide if the predicate:

(5) $$P(X,S)=\underset{1\leq i\leq n,1\leq k\leq r}{\wedge }P_{i,k}(X,S)[x^{k}]$$

can be satisfied. If so, find all variables that take the same value in all admissible valuations (so called *hard components *of the system).

### Decision diagram encoding

In a previous work [26], we showed how the set of solutions of a qualitative system can be computed as a decision diagram [37]. A decision diagram is a data structure meant to represent functions on finite domains; it is widely used for the verification of circuits or network protocols. Using such a compact representation of the set of solutions, we proposed efficient algorithms for computing solutions of the systems, hard components, and other properties of a qualitative system. Back to our problem: in order to predict the regulatory role of TFs on their target genes, it is enough to compute the decision diagram representing the predicate (5), and compute its hard components as proposed in [26]. This approach is suitable for systems of at most a couple of hundred variables. Above this limit, the decision diagram is too large in memory complexity. In our case however, we consider systems of about 4000 variables at most, which is far too large for the above mentioned algorithms.

In order to cope with the size of the problem, we propose to investigate a particular case, when all species are observed, in all experiments. In this case, *i *≠ *j *implies that *P*_*i*, *k*_(*X*, *S*) [*x*^*k*^] and *P*_*j*, *k*_(*X*, *S*) [*x*^*k*^] share no variables. This means that *P *may be satisfied if and only if each predicate

(6) $$P_{i,.}(S)=\underset{1\leq k\leq r}{\wedge }\exists X\;P_{i,k}(X,S)[x^{k}]$$

may be satisfied. As a consequence, a variable *S*_*ji *_is a hard component of *P *if and only if it is a hard component of *P*_*i*,.. _*P*_*i*,. _correspond to the constraints which relate species *i *to its predecessors in *G *for all experiments. The number of variables in *P*_*i*,. _is exactly the in-degree of species *i *in *G*, which is at most 10–20 in biological networks.

As soon as some species are not observed in some experiment, the predicates *P*_*i*,. _share some variables and it is not guaranteed to find all hard components by studying them separately. A brief investigation showed (data not shown) that due to the topology of the graph, most of the equations are not independent any more, even with few missing nodes. Note however, that any hard component of *P*_*i*,. _is still a hard component of *P*. The same statement holds for

(7) $$P_{.,k}(X)=\underset{1\leq i\leq n}{\wedge }\exists S\;P_{i,k}(X,S)[x^{k}]$$

where *P*_.,*k *_corresponds to the constraints that relate all species in *G *for a *single *experiment. Relying on this result, we implemented the following algorithm

In practice, this algorithm is very effective in terms of computation time and number of hard components found. However, as already stated, it is not guaranteed to find all hard components of *P*. This is what motivates the technique described in the next paragraph.

### Solving with Answer Set Programming

In order to solve large qualitative systems, we also tried to encode the problem as a logic program, in the setting of answer set programming (ASP). While decision diagrams represent the set of *all *solutions, finding a model for a logic program provides *one *solution. In order to find hard components, it is enough to check for each variable *V*, if there exists a solution such that *V *= + and another solution such that *V *= -. The ASP program we used in order to solve the qualitative system is given in supplementary materials. In the following we will denote by *asp_solve*(*P*) the call to the ASP solver on the predicate *P*. The returned value is an admissible valuation if there is one, or ⊥ otherwise. The complete algorithm is reported below

We use clasp for solving ASP programs [38], which performs astonishingly well on our data. The procedure described in Algorithm 3 is particularly efficient in finding *non *hard components: generating one solution may be enough to prove non hardness of many variables at a time.

To sum up, in order to solve a system of qualitative equations (4) with only partial observations, we use Algorithm 2 first and thus determine most (if not all) hard components. Then, Algorithm 3 is used for the remaining components, which are nearly all non hard.

### Reduction technique

As mentioned in the Result section, interaction graphs may be reduced in a way that preserves the satisfiability of the associated qualitative system. Consider a graph *G *with defined signs on its edges. If some node *n *has no successor, then deletes it from *G*. Note then, that any solution of the qualitative system associated to the new graph can be extended in a solution to the system associated to *G*. The same statement holds if one iteratively delete all nodes in the graph with no successor. The result of this procedure is the subgraph of *G *such that any node is either on a cycle, or has a cycle downstream. We refer to it as the core of the interaction graph.

The core of an interaction graph corresponds to the most difficult part to solve, because extending a solution for the core to the entire graph can be done in polynomial time, using a breadth-first traverse.

### Diagnosis for noisy data

When working with real-life data, it may happen that the predicate *P *defined in Eq. (5) cannot be satisfied. This may be due to three (non exclusive) reasons:

• a reported expression data is wrong

• an arrow (or more generally a subgraph) is missing

• the sign on an edge depends on the state of the system

In the third case, the conditions for deriving Eq. (1) are not fulfilled for one node and its qualitative equation should be discarded. This, however, does not affect the validity of the remaining equation.

In all cases, isolating the cause of the problem is a hard task. We propose the following diagnosis approach: as *P *is a conjunction of smaller predicates, it might happen that some subsets of the predicates are not satisfiable yet. Our strategy is then to find "small" subsets of predicates which cannot be satisfied. A particularly interesting feature of this approach is that by selecting subsets of *P*_*i*,. ,. _predicates, the result might directly be interpreted and visualised as a subgraph of the original model.

### How to determine if a sign can be inferred

In the Results section, we have seen some examples showing that even when all feasible observations are available, it might not be possible to infer all signs in the interaction graph. Whether or not a sign can be inferred depends on the topology of the graph, and also on the actual signs on interactions. In practice, it is thus impossible to tell from the unsigned graph only if a sign can be recovered. However, it is still interesting to evaluate on fully signed interaction networks which part can be inferred. A trivial algorithm for this consists in explicitly generating all feasible observations and using the algorithms described above. This is unfeasible due to the number of observations.

With the notations introduced above, consider an observation *X *and sign variables *S *for an interaction graph. *P*_*i*_(*X*, *S*) denotes the constraints that link the variation of a node *i *to that of its predecessors given the signs of the interactions. Moreover, the real signs in the graph are denoted by *s*. For each node *i*, we build the predicate giving the feasible observations on node *i *and its predecessors, given the rest of the graph and the real signs *s*

$$O_{i}(X)=\exists X_{j_{j\in \{i\}\cup \text{pred}(i)}}\underset{1\leq i\leq n}{\wedge }P_{i,k}(X,s)$$

Then, the constraint that we can derive on *S *variables is: for any observation *X *that is feasible *P*_*i*_(*X*, *S*) should hold. This constraint is more formally defined by

*C*_*i*_(*S*) = ∀*XO*_*i*_(*X*) ⇒ *P*_*i*_(*X*, *S*)

Finally, the hard components of *C*_*i *_are exactly the signs that can be inferred using *all *feasible observations. Let us sum up the procedure:

1. compute *P*(*X*, *S*) = ∧_1 ≤ *i *≤ *n *_*P*_*i*_(*X*, *s*)

2. compute *O*_*i *_from *P *and the actual signs *s*

3. compute *C*_*i*_, the constraints of signs given all feasible observations

4. compute the hard components of *C*_*i*_, which are exactly the signs that can be inferred.

If it is not possible to compute *P*(*X*, *S*) (mainly because the interaction graph is too large), we use a more sophisticated approach based on a modular decomposition of the interaction graph. The resulting algorithm, as well as all inference algorithms, experimental data, and the results obtained for the *S. cerevisiae*, and *E. coli *regulatory networks can be found at: .

## Authors' contributions

PV participated in designing the algorithms described in the Methods section and in performing the simulations. CG designed the algorithms described in the Results section, and performed the analysis on *E. coli *and yeast data. OR made the statistical modeling in the Results section. MLB participated in implementing the algorithms on decision diagrams. All authors participated in writing the manuscript, read and approved its final state.

## Appendix

Algorithm 1

Naive Inference algorithm

   **Algorithm: **Naive Inference algorithm

   **Input:**

         a network with its topology

         a set of expression profiles

   **Output:**

         a set of predicted signs

         a set of ambiguous interactions

   **For all **Node *A *with exactly one predecessor *B*

   **if ***A and B are observed simultaneously ***then return**

      *prediction sign*(*B *→ *A*) = *sign*(*A*) * *sign*(*B*)

   **if ***sign*(*B *→ *A*) *was predicted different by another*

   *expression profile **then return **Ambiguous arrow B *→ *A*

Algorithm 2

Heuristic for finding hard components in large interaction networks with many expression profiles.

   **Input:**

      the predicates *P*_*i*,. _and *P*_.,*k *_for all *i *and *k*

      observed variations *x*

   **Output:**

      a set *s *of hard components of *P*

   *s *← ∅

   **while ***True ***do**

      *s' *← ∪_*i *_*hard_components*(*P*_*i*,. _[*x*^*k*^, *s*])

      **if ***s' *= ∅ **then return ***s*

      *s *← *s *∪ *s'*

      *x' *← ∪_*k *_*hard_components*(*P*_.,*k *_[*x*^*k*^, *s*])

      **if ***x' *= ∅ **then return ***s*

      *x *← *x *∪ *x'*

   **end**

Algorithm 3

Exact algorithm for finding the set of hard components of *P*, based on logic programming.

   **Algorithm:**Hard components using ASP

   **Input:**

         the predicates *P*

         observed variations *x*

   **Output:**

         a set *h *of hard components of *P*

   *h *← ∅

   *C *← {*S*_*ji*_|*j *→ *i*}

   s * ← *asp_solve*(*P*)

   **if **s* = ⊥ **then return **⊥

   **while ***C *≠ ∅ **do**

      choose *V *in *C*

      *s *← *asp_solve*(*P *[*V *= $-s_{V}^{*}$])

      **if ***s *= ⊥ **then**

         *h *← {(*V*, *s*_*V*_)} ∪ *h*

      **else**

         delete from *C *all *W *in *C *s.t. any $s_{W}^{*}$ ≠ = *s*_*W*_

      **end**

   **end**
